# Chemical Cell Lysis with Clarification Filtration of Suspension Cell Culture-Derived Modified Vaccinia Virus Ankara

**DOI:** 10.3390/vaccines14060468

**Published:** 2026-05-25

**Authors:** Linus G. Weber, Larissa Dörr, Caroline Stephan, Leon Freitag, Leander John, Ingo Jordan, Michael W. Wolff

**Affiliations:** 1 Centre of Competence for Biotechnology and Biomedical Physics (BioTechMed), Institute of Bioprocess Engineering and Pharmaceutical Technology, University of Applied Sciences Mittelhessen (THM), Wiesenstr. 14, 35390 Giessen, Germany; linus.georg.weber@lse.thm.de (L.G.W.);; 2 ProBioGen AG, Goethestr. 54, 13086 Berlin, Germany

**Keywords:** cell lysis, clarification, detergent, modified vaccinia virus ankara, poxvirus, vaccine manufacturing, viral vector

## Abstract

**Background**: Modified Vaccinia Ankara (MVA) vectors are highly immunogenic vaccine platforms for the delivery of recombinant antigens. Efficient downstream processing is still challenging, particularly because substantial fractions of the virus remain intracellular. While chemical cell lysis that releases MVA particles into the supernatant before clarification can greatly enhance process efficiency and scalability, this step remains insufficiently characterized. **Methods**: This study assessed the compatibility of ionic, non-ionic, and zwitterionic detergents with the virus as purification target. Polysorbate 20 (Tween 20) was selected as a candidate detergent and evaluated across harvest times of 48–72 h post-infection (hpi) at concentrations of 0.01–0.5% (*v*/*v*). **Results**: The addition of 0.01% to 0.05% Tween 20 at 48 hpi resulted in a twofold increase in supernatant virus within one hour of application. Extended exposure to Tween 20, combined with a 650 mM mixture of NaCl, NaBr, and KCl, promoted virus particle release. However, Tween 20 concentrations above 0.1% reduced MVA infectivity. A filtration cascade using pore sizes of 5 µm and 1.2 µm achieved product yields of 77–83% at 48 hpi and 41–69% at 72 hpi, respectively. Host-cell DNA is an important contaminant during viral vector processing. However, the application of 0.05% (*v*/*v*) Tween 20 resulted in a 35% reduction of dsDNA released into the culture supernatant; the nuclei could not be preserved intact under high-salt conditions to avoid the release of cellular DNA. **Conclusions**: In summary, this comprehensive data demonstrated that non-ionic detergents can be used to induce cell lysis while maintaining infectious activity of enveloped MVA.

## 1. Introduction

Members of the family Poxviridae are large, enveloped viruses that replicate within the cytoplasm and must be released from the host cell prior to purification [1,2]. Modified Vaccinia virus Ankara (MVA, genus Orthopoxvirus) is a highly attenuated poxvirus under consideration as a vaccine vector in preclinical and clinical studies against infectious [3,4,5,6,7,8,9] and neoplastic diseases [10,11]. Attenuation of MVA has been achieved by serial passaging of a vaccinia virus in primary chicken cells and is based on the loss of host range factors and accessory genes [6,12,13,14,15]. Because MVA cannot replicate in human cells, this virus is safe even for immunocompromised patients, but it has to be given at a high 10^8^ infectious units per dose [16].

The morphogenesis of vaccinia viruses results in distinct infectious forms: intracellular mature, intracellular wrapped (particles with three membranes derived from the trans-Golgi network), cell-associated enveloped, and extracellular enveloped viruses [17,18,19,20]. The third membrane of the wrapped viruses fuses with the plasma membrane for egress and is left behind, leaving a doubly enveloped particle for the spread of infectious units in the host organism. The second membrane of extracellular enveloped viruses must be disrupted to expose the mature (singly enveloped) virions as the true infectious unit [21].

However, only a fraction of the intracellular, mature virions proceed toward the plasma membrane and are released. Replication of vaccinia viruses is, furthermore, not accompanied by extensive host cell lysis, and a large fraction of virus progeny remains intracellular or cell-associated after virus assembly [17,22]. Therefore, achieving the complete release of the intracellular virus from the host cell is important to maximize overall process yield.

Since infectious activity is crucial for the therapeutic efficacy, the cell lysis method must preserve virion integrity and avoid interference with downstream processing (DSP) or analytical assays. Reported methods to mechanically break up cells after MVA infection include sonification and repeated freeze–thaw cycles. However, both are limited to smaller-scale applications [23,24,25,26]. The facilitated transfer to large-scale production is a key advantage of chemical agents for cell lysis. Detergents are amphipathic, consisting of both hydrophilic and hydrophobic regions. They solubilize the lipid bilayer of the cell membrane and disrupt the lipid–lipid, lipid–protein, and protein–protein interactions, creating pores that lead to cell lysis and release of viral vectors [27,28]. Based on their charge, detergents are classified into cationic, anionic, zwitterionic, or non-ionic [29]. The type and concentration of detergent significantly affect membrane solubilization dynamics and the overall extent of lysis. Ionic detergents, such as sodium dodecyl sulfate (SDS), are potent agents that can rapidly disrupt membranes and solubilize proteins, but they also tend to denature proteins [30]. Zwitterionic detergents, such as 3-[(3-cholamidopropyl)dimethylammonio]-1-propanesulfonate (CHAPS), contain both negatively and positively charged groups but have a net neutral charge [31,32]. Similar to ionic detergents, they effectively break protein–protein bonds, but their effect on the protein structure is typically less harsh. Non-ionic detergents such as polysorbate 20 (Tween 20) or Triton X-100 are milder agents that can be used to lyse cells while preserving protein structure and biological activity, though they may require longer incubation times or higher concentrations [33]. For a vaccine production process to be compliant with modern standards, detergents must be listed under the Registration, Evaluation, Authorization, and Restriction of Chemicals (REACH) regulation.

The efficiency of chemical lysis depends furthermore on its concentration, and environmental factors, such as temperature, salt concentration, and the presence of cellular components like lipids, proteins, and nucleic acids [34,35]. With respect to viral vectors, detergents were successfully used in the processing of the non-enveloped adeno-associated virus (AAV), where Triton X-100, Triton-CG110, and Tween 20 have demonstrated a complete lysis of human embryo kidney (HEK293) cells [28,34,36]. Low concentrations of Tween 20 have been reported to increase virus titers during the infection of PK-15 cells with (the also non-enveloped) porcine circovirus 2 [37]. In cell culture-derived vaccine production, detergents are applied to large-scale bioreactor cultures after vector amplification to release intracellular product fractions and enhance yields during DSP. However, the choice of detergent and environmental factors needs to be carefully adjusted to protect the lipid bilayer of enveloped viruses [38], and to our knowledge, chemical cell lysis has not yet been reported for poxviruses.

We demonstrate that detergent-based lysis can facilitate process scalability and maximize overall yields. Various detergents were screened to maximize the release of intracellular viral particles, while preserving their infectivity. A key focus is optimizing lysis conditions to facilitate the DSP. Specifically, this method aimed to maintain the structural integrity of host cell nuclei to improve contaminant removal via dead-end filtration, thereby increasing overall process yields and purity for clinical-grade vaccine manufacturing.

## 2. Materials and Methods

### 2.1. Materials and Reagents

The avian cell line AGE1.CR.pIX and the virus strain MVA-CR19.GFP were kindly provided by ProBioGen AG (Berlin, Germany). The CD-U7 cell culture media was purchased from Sartorius Xell GmbH (Bielefeld, Germany), and the basal Dulbecco’s Modified Eagle Medium (DMEM) with (L0104-500) and without sodium pyruvate (L0102-500) were purchased from Biowest (Nuaillé, France). Adherent Vero cells (CCL-81) were from American Type Culture Collection (ATCC, Manassas, VA, USA). The media supplement fetal bovine serum (FBS) was obtained by Capricorn Scientific GmbH (Dreihausen, Germany), the recombinant insulin-like growth factor (LONG^®^R^3^IGF-I, 10-1011-5) from Repligen (Waltham, MA, USA), and the GlutaMAX™ from ThermoFisher Scientific (Waltham, MA, USA). The trypsin and phosphate-buffered saline (PBS) were purchased from Sigma Aldrich (Merck KGaA, Darmstadt, Germany). The cell culture plasticware was purchased from Sarstedt AG & Co. KG (Nümbrecht, Germany), and the 24-well plates from Greiner Bio-One International GmbH (Kremsmuenster, Austria). The detergents used in this study, including polysorbate 20 (Tween 20, P2287-100ML) and Zwittergent 3–14 (693017-5GM) were purchased from Merck KGaA (Darmstadt, Germany). Polysorbate 80 (Tween 80, MB301-100G) was purchased by HiMedia Laboratories GmbH (Einhausen, Germany), CHAPS (17038.02) from SERVA Electrophoresis GmbH (Heidelberg, Germany), and sodium deoxycholate (SDC, A1531) from AppliChem GmbH (Darmstadt, Germany). For clarification, a 5.0 µm syringe filter (Minisart^®^ NML Syringe Filter, 6.2 cm^2^ total filtration area, S7594-FMOSK) and a 1.2 µm syringe filter (Minisart^®^ NML Syringe Filter, 6.2 cm^2^ total filtration area, 17593-K) were purchased from Sartorius AG (Goettingen, Germany). All other chemicals were obtained by Carl Roth GmbH & Co. KG (Karlsruhe, Germany) in analytical grade, unless otherwise stated.

### 2.2. MVA Vector Production

The suspension cell line AGE1.CR.pIX [24] was cultivated in chemically defined CD-U7 medium, supplemented with 2 mM GlutaMAX™ and 10 ng/mL recombinant insulin-like growth factor. The cells were inoculated at 0.8–1.0 × 10^6^ cells/mL in baffled glass shake flasks of 100–1000 mL with 0.2 µm vent caps. Cultures were maintained at 37 °C and 8% CO_2_ in air and 180 rpm under orbital shaking (25 mm shaking diameter, Infors HT) [39]. The virus used was the recombinant strain MVA-CR19.GFP encoding a green-fluorescent-protein insertion cassette [22]. For viral amplification, cultures were infected with a multiplicity of infection (MOI) of 0.05 at a density of 2.0 × 10^6^ cells/mL in a 1:1 mixture of CD-U7 and basal DMEM containing sodium pyruvate [22].

### 2.3. Detergent Screening for an Optimal Cell Lysis Agent

For detergent screening, 5% (*w*/*v*) sterile-filtered stock solutions were prepared for Tween 20, Tween 80, Zwittergent™ 3–14, CHAPS, and SDC. At 48 h post-infection (hpi), chaotropic salts were added to the culture to achieve a final concentration of 250 mM NaCl, 250 mM NaBr, and 150 mM KCl to prevent adsorption of DNA fragments on enveloped virus particles [40]. Taking the medium composition into account, the total salt concentration was estimated to be 900 mM. In biological triplicates, 1.5 mL samples from 72 hpi harvest were incubated for 1 h at 37 °C and 350 rpm (Thermomixer Comfort; Eppendorf, Hamburg, Germany) with detergent concentrations of 0.0005%, 0.005%, 0.05%, and 0.5% (*v*/*v*). Following incubation, the triplicate samples were pooled and analyzed directly for remaining intact cells or centrifuged at 200× *g* for 5 min. As a reference treatment, the infected cell culture was subjected to three freeze–thaw cycles (−80 °C) and sonification at 30 Ws/mL (Bandelin with K76 probe) for complete cell lysis. Supernatants were collected by centrifugation at 200× *g* for 5 min [41]. All supernatants were diluted 1:1000 in DMEM supplemented with 5% FCS and stored at −80 °C for infectious virus titer analysis.

### 2.4. Determination of Tween 20 Concentration

To investigate the effect of Tween 20 concentrations, cells were infected as described in Section 2.2, and chaotropic salts were added at 48 hpi (Section 2.3). Following 1 h of incubation under high ionic strength conditions, final concentrations of 0%, 0.01%, 0.05%, 0.5%, 1.0%, and 2.5% (*v*/*v*) Tween 20 were added to the virus harvest in 1.5 mL sampling tubes and shaken at 350 rpm. After an additional 1 h of incubation, the supernatants were collected by centrifugation and stored as described in Section 2.3.

### 2.5. Tween 20 Treatment in Shake Flasks

The most promising Tween 20 concentrations were compared and evaluated in a scalable shake flask setup. Infected cells were harvested at 48 hpi and 72 hpi, with the addition of chaotropic salts (Section 2.2 and Section 2.3). After 1 h of incubation under high ionic strength conditions, Tween 20 was added to shake flasks to achieve final concentrations of 0%, 0.01%, 0.05%, 0.1%, 0.25%, and 0.5%. Samples (1 mL) were collected at 0, 1, 2, 4, and 6 h post-detergent addition. These samples were analyzed directly in triplicate for the total concentration of remaining intact cells. The supernatant was then collected via differential centrifugation at 200× *g* and 4600× *g* for 5 min. Subsequently, the samples were stored as described in Section 2.3 until further use.

### 2.6. Clarification Filtration

The virus harvested from Section 2.5 was clarified after 8 h of incubation with Tween 20. For the clarification process, 6 mL of harvest was filtered through a 5.0 µm syringe filter, and 4 mL of the resulting filtrate was passed through a 1.2 µm syringe filter. Prior to use, each filter was equilibrated with 2 mL of buffer (20 mM Tris, 250 mM NaBr, 250 mM NaCl, 150 mM KCl, pH 7.4). The clarified samples were diluted and stored as described in Section 2.3 until further use.

### 2.7. Effect of Temperature and Tween 20 Concentration on Infectivity in the Virus Harvest

To determine the stability of the viral infectivity within the harvest matrix, a 48 hpi harvest containing 250 mM NaBr, 250 mM NaCl, and 150 mM KCl was used. The harvest was aliquoted into 1.5 mL sampling tubes and adjusted to final concentrations of 0%, 0.01%, 0.05%, 0.1%, and 0.5% Tween 20 (*v*/*v*). All samples were shaken at 350 rpm, incubated at 4 °C, 21 °C, and 37 °C, and sampled after 0, 4, and 8 h. To assess temperature effects, Tween 20 was fixed at 0.05%, and samples at 4 h and 21 °C were taken in triplicate, while single samples were collected at 4 °C, 37 °C, 0 h, and 8 h. The impact of the detergent concentration was investigated by keeping the temperature constant at 21 °C. The previously mentioned Tween 20 concentrations and incubation times were sampled in biological triplicates. All samples were subsequently diluted and stored as described in Section 2.3.

### 2.8. Cell Concentration and Viability

Differentiation of live and dead cells was done by fluorescence-activated cell sorting (FACS), with propidium iodide (PI) staining, using the Guava^®^ easyCyte™ HT flow cell cytometer (Cytek Biosciences B.V., Fremont, CA, USA) [42]. Cells were diluted 1:10 or 1:20 in 0.005 g/L propidium iodide in PBS with a final volume of 200 µL, immediately before analysis. The light scattering profiles and fluorescence intensities were determined. Measurements were acquired using a Red-B fluorescence filter, as propidium iodide is excited at a wavelength of 488 nm and emits at a wavelength of 617 nm.

### 2.9. Flow Cytometric Virus Titration

Quantification of infectious MVA particles was performed by detection of GFP expression in Vero cells via flow cytometry. Cells were seeded in 24-well plates, with 100,000 cells per well in a culture volume of 1 mL DMEM supplemented with 5% FCS. Infections were performed in triplicate by adding 100 µL of sample, medium blank, or standard virus stock (ranging from 1.6 × 10^4^ TCID_50_/mL to 1.0 × 10^6^ TCID_50_/mL). After incubation for 16–18 h at 37 °C and 8% CO_2_, the cell layer was washed twice with PBS. The cells were detached with Trypsin/EDTA, collected by centrifugation at 400× *g* for 2 min, resuspended in 150 µL of PBS, and analyzed by flow cytometry for the percentage of GFP-positive (equal to infected) cells compared to the total cell number. The infectious titer, expressed in TCID_50_/mL, was calculated using a standard curve derived from a known MVA reference standard. The interassay coefficient of variation of the method as applied here was ≤20%, the lower limit of detection was 3.1 × 10^4^ TCID_50_/mL, and the lower limit of quantification was 5.1 × 10^4^ TCID_50_/mL.

### 2.10. DNA Cell Nucleus Staining

To visualize host cell DNA, 1 mL of pretreated virus harvest was centrifuged at 4600× *g* for 5 min. The cell pellet was washed three times with PBS and incubated with Hoechst 33,342 to a final concentration of 16 µM for 5 min. The pellet was washed twice with PBS and resuspended in 2 mL PBS, transferred to a 6-well plate, and imaged using a multimode imaging reader (BioTek™ Cytation 3™, Fisher Scientific, Waltham, MA, USA).

### 2.11. Total dsDNA Quantification

The concentration of double-stranded DNA (dsDNA) was determined using the Quant-iT™ PicoGreen™ dsDNA Assay Kit (Thermo Fisher Scientific, Waltham, MA, USA) according to the manufacturer’s instructions as previously described [43]. Briefly, samples were diluted 1:400 in the assays’ 1 ×TE buffer. In a black 96-well plate (Nunc, Thermo Fisher Scientific, Waltham, MA, USA), 100 µL of the diluted sample was mixed with 100 µL of the Picogreen™ dye reagent. The plate was analyzed for fluorescence emission at 520 nm after an excitation at 480 nm using a multimode imaging reader (BioTek™ Cytation 3™, Fisher Scientific). Final concentrations were calculated from standard calibrations ranging from 1–1000 ng/mL. Standards were freshly prepared for each plate, and the assay standard deviations across technical triplicates were below 4%.

## 3. Results

### 3.1. Detergent Screening

#### 3.1.1. Mechanical Lysis Comparison

**Figure 1:**
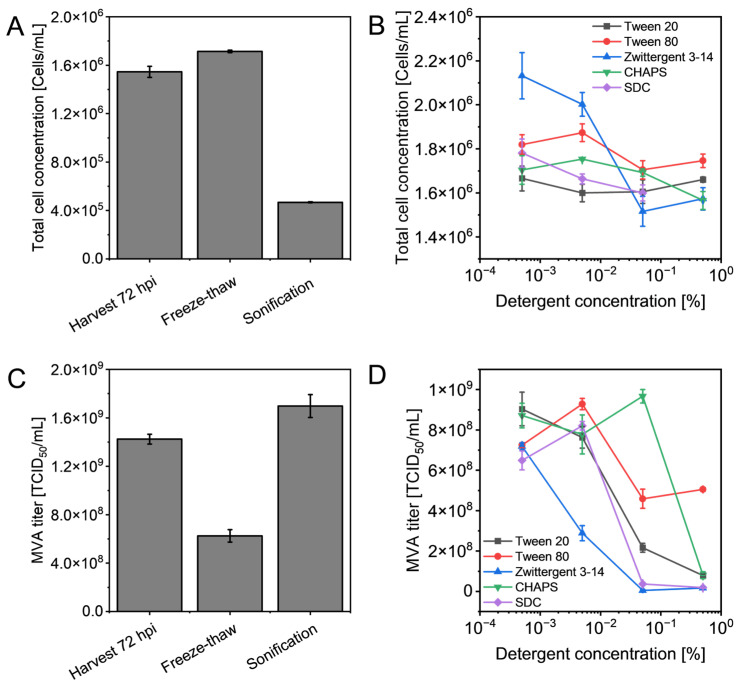
Total cell concentrations and infectious virus titers in supernatant after treatment of AGE1.CR.pIX cells infected with MVA-CR19.GFP using five candidate detergents, compared with repeated freeze–thaw cycles and sonification as reference lysis methods. (**A**) Total cell concentration after reference treatments, (**B**) total cell concentration after treatment by detergents, (**C**) infectious virus titers in references, and (**D**) infectious virus titers in culture supernatants containing detergents. Error bars represent standard deviations of technical triplicates from three pooled samples.

First, a screening of various zwitterionic, non-ionic, and ionic detergents was performed to assess their impact on remaining intact cells and MVA infectivity. At the time of harvest (TOH) 72 hpi, the untreated control exhibited a total cell concentration of roughly 1.5 × 10^6^ cells/mL and an infectious virus titer of 1.4 × 10^9^ TCID_50_/mL. Three repeated freeze–thaw cycles (−80 °C to 21 °C) did not result in cell disruption but led to a 57% decrease in infectious MVA titers (from 1.4 × 10^9^ TCID_50_/mL to 6.0 × 10^8^ TCID_50_/mL). In contrast, sonification reduced the cell concentration from 1.5 × 10^6^ cells/mL to 0.5 × 10^6^ cells/mL (Figure 1A) and was associated with a 21% increase in the infectious titer by nearly 3.0 × 10^8^ TCID_50_/mL (from 1.4 × 10^9^ TCID_50_/mL to 1.7 × 10^9^ TCID_50_/mL, Figure 1C), compared with the non-treated samples.

#### 3.1.2. Detergent Effects on Cell Integrity

The zwitterionic detergents, Zwittergent 3–14 and CHAPS, reduced the total cell concentration in a dose-dependent manner (Figure 1B). Increasing the concentration from 0.0005% to 0.5% resulted in a cell loss due to disruption down to 0.5 × 10^6^ cells/mL for Zwittergent 3–14 and 0.1 × 10^6^ cells/mL for CHAPS. For the anionic detergent SDC, a maximum cell reduction of 0.2 × 10^6^ cells/mL was observed by increasing the concentration from 0.0005% to 0.05%. The concentration of 0.5% SDC was not measurable due to its high viscosity. Using Tween working concentrations, ranging from 0.0005% to 0.5%, led to a maximal reduction of 0.1 × 10^6^ cells/mL for Tween 80, while no reductions were detected in the presence of Tween 20.

#### 3.1.3. Detergents’ Effect on Virus Infectivity

The critical micelle concentration (CMC) is the specific concentration of detergent above which micelles begin to form. Additional detergent may largely be bound into micelles, while the concentration of free detergent monomers remains approximately constant. Since membrane disruption depends on the concentration of free detergent, detergent concentrations close to or above the CMC are typically required to solubilize plasma membranes [44,45]. As the CMC varies with the composition of the detergent, the working ranges were individually determined. Zwittergent 3–14, CHAPS, and SDC have a higher CMC than Tween 20 and 80, while higher required concentrations are expected for sufficient cell break-up [46,47,48]. At a CMC of 0.05% Zwittergent 3–14, 0.5% CHAPS, and 0.5% SDC, the virus infectivity dropped below 1 × 10^8^ TCID_50_/mL. The peak virus titers in supernatants for these detergents could be achieved below the CMC for 0.005% SDC and 0.05% CHAPS until viral infectivity was lost.

The highest virus infectivity in supernatant was achieved for 0.005% Tween 80 with 9.0 × 10^8^ TCID_50_/mL (Figure 1D). The closest tested concentration to the CMC of Tween 20 was 0.005% (*v*/*v*), which resulted in an infective virus titer of 7.6 × 10^8^ TCID_50_/mL, while the CMC is estimated to be 0.006% (*w*/*w*) [49]. Compared to a non-supplemented control, the virus titers in supernatant containing 0.005% Tween 80 or Tween 20 revealed a loss of infectious MVA of 5.0 × 10^8^ TCID_50_/mL and 6.4 × 10^8^ TCID_50_/mL, respectively. In summary, the data indicate that exceeding the CMC leads to a marked loss of viral infectivity, highlighting the importance of free detergent monomer concentration in balancing virus release and stability.

### 3.2. Influence of Tween 20 Concentration on MVA Release and Infectivity

**Figure 2:**
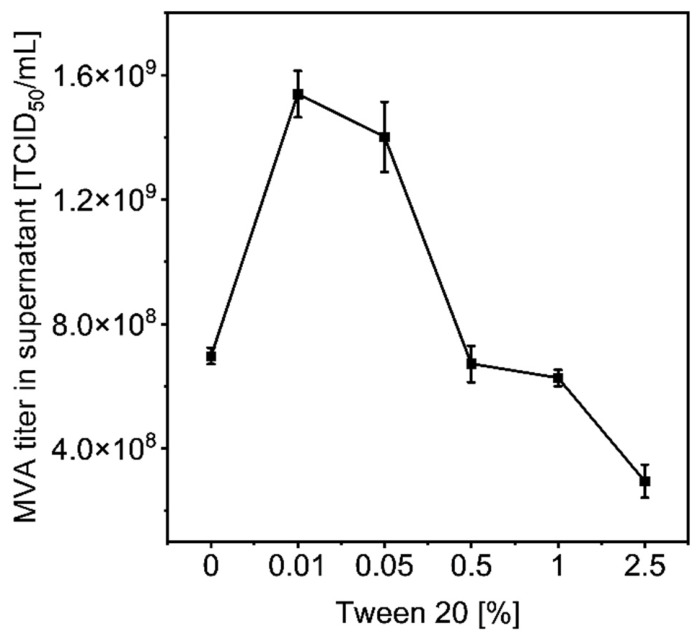
Influence of Tween 20 concentration on infectious MVA-CR19.GFP titers. AGE1.CR.pIX cells were harvested at 48 hpi, pretreated with 250 mM NaCl, 250 mM NaBr, and 150 mM KCl, before incubation for 1 h with Tween 20 (0–2.5% (*v*/*v*)). Infectious titers in the supernatant were determined via flow cytometry and expressed as TCID_50_/mL. Values represent the mean of technical triplicates ± standard deviation.

The impact of varying Tween 20 concentrations above the CMC on MVA release was evaluated at 48 hpi, as described in Section 2.4. The addition of 0.01% and 0.05% Tween 20 resulted in increased infectious MVA virus titers of 8.0 × 10^8^ TCID_50_/mL and 7.0 × 10^8^ TCID_50_/mL, compared to a non-treated control (Figure 2). Within 1 h of incubation in Tween 20, the amount of infective MVA particles in the supernatant roughly doubled to a virus titer of 1.5 × 10^9^ TCID_50_/mL, but concentrations ≥ 0.5% Tween 20 led to MVA infectivity reduction compared to the non-treated control.

### 3.3. Cell Lysis Investigation in Scalable Shake Flasks at Different Times of Harvests

To analyze cell lysis in a scalable experimental design, experiments were upscaled to shake flasks using a Tween 20 concentration range of 0.01% to 0.5%, at two TOH (48 hpi and 72 hpi), with incubation times ranging from 0 to 6 h.

#### 3.3.1. Viral Infectivity and Release at 48 hpi

**Figure 3:**
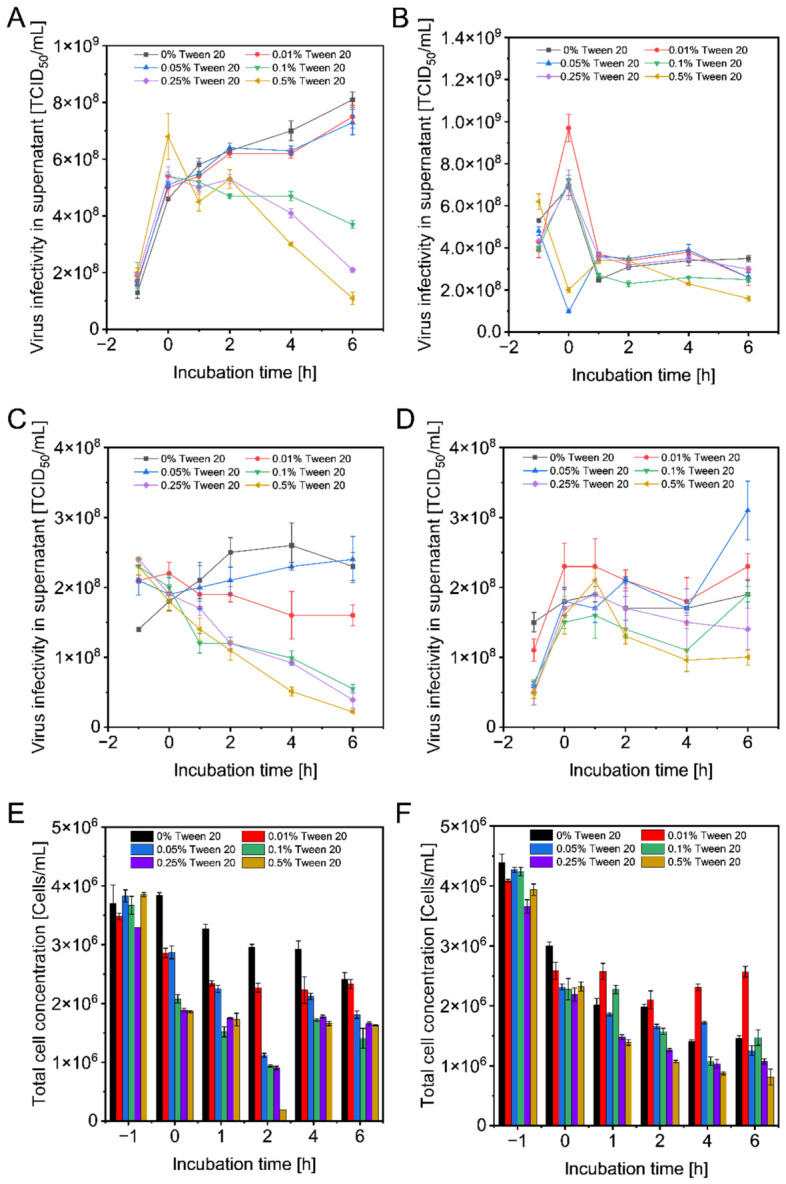
Comparison of lysis of AGE1.CR.pIX cells infected with MVA-CR19.GFP at 48 hpi and 72 hpi, using different Tween 20 concentrations. The chaotropic salts, 250 mM NaCl, 250 mM NaBr, and 150 mM KCl, were added at −1 h and the Tween 20 at 0 h of incubation time. (**A**) Virus in supernatant after centrifugation of 48 hpi at 200× *g* for 5 min. (**B**) Virus in supernatant after centrifugation of 72 hpi at 200× *g* for 5 min. (**C**) Virus in supernatant after centrifugation of 48 hpi at 4600× *g* for 5 min. (**D**) Virus in supernatant after centrifugation of 72 hpi at 4600× *g* for 5 min. (**E**) Total cell concentration after 48 hpi. (**F**) Total cell concentration after 72 hpi. The bars represent means of technical triplicates and their respective standard deviations.

At 48 hpi, the addition of mild chaotropic salts in the non-treated control (0% Tween 20) resulted in an increase of infectious virus in the supernatant, from 1.3 × 10^8^ TCID_50_/mL to 4.6 × 10^8^ TCID_50_/mL after 1 h of incubation, and to 8.1 × 10^8^ TCID_50_/mL after 6 h of incubation (Figure 3A). Supernatants treated with Tween 20 showed an initial increase of at least 0.4 × 10^8^ TCID_50_/mL in infective MVA titer associated with increasing Tween 20 concentration (Table 1). With the initial addition of Tween 20, the concentration of 0.5% showed an increase of virus in supernatant directly after detergent addition, by approximately 2.2 × 10^8^ TCID_50_/mL, compared to the non-treated control. This corresponds to an increase of 48% of infective MVA particles in supernatant compared to the non-treated control. With advanced incubation time to 6 h, Tween 20 concentrations up to 0.05% showed no virus accumulation in supernatant, compared to the non-treated control. The virus was inactivated with Tween 20 concentrations exceeding 0.05%. After 6 h of incubation in 0.5% Tween 20, a reduction from roughly 6.8 × 10^8^ TCID_50_/mL to 1.1 × 10^8^ TCID_50_/mL was observed.

Next, virus harvests were centrifuged at 4600× *g* to separate the virus particles from cells and cell debris (Figure 3C). The control of the supernatant without Tween 20 revealed an increase of the virus from 1.4 × 10^8^ TCID_50_/mL to 1.8 × 10^8^ TCID_50_/mL after 1 h incubation, and an increase to 2.3 × 10^8^ TCID_50_/mL after 6 h of incubation.

Concentrations of 0.01% and 0.05% Tween 20 led to no increase of virus in 4600× *g* supernatant, compared to the non-treated control (Figure 3C). After 6 h of incubation with Tween 20, at concentrations of 0.1–0.5%, increasing detergent concentration correlated with infectivity loss of extracellular virus. The observed span (approximately 0.3 × 10^8^ TCID_50_/mL) was smaller compared with the span of 3.0 × 10^8^ TCID_50_/mL reported in Figure 3A, when 200× *g* centrifugation was applied.

When the virus was harvested after 48 hpi, a total cell concentration of 3.8 × 10^6^ cells/mL was achieved (Figure 3E). Compared to the cell concentration of 2.0 × 10^6^ cells/mL at the time of infection, the counted cells increased by a factor of 1.9 during the infection phase. Following 6 h of incubation in mild chaotropic salts, the cell concentration was reduced by 1.3 × 10^6^ cells/mL (from 3.7 × 10^6^ cells/mL to 2.4 × 10^6^ cells/mL). Addition of 0.01% Tween 20 for 1 h of incubation led to a higher reduction of total cell concentration (1.2 × 10^6^ cells/mL) compared to the control, with a reduction of 0.5 × 10^6^ cells/mL. The final cell concentration after 6 h of incubation in 0.01% Tween 20 was not affected compared to the untreated control. Higher detergent concentration between 0.05–0.5% Tween 20 led to cell break-up with a reduction of 1.6–2.3 × 10^6^ cells/mL, which corresponds to 25–48% higher cell reduction compared to the non-treated control (Figure 3E).

#### 3.3.2. Viral Infectivity and Release at 72 hpi

To study the cell lysis with cultures at a more advanced state of infection, the virus harvest at 72 hpi was treated. The control showed an increase in the virus titer of 1.6 × 10^8^ TCID_50_/mL (from 5.3 × 10^8^ TCID_50_/mL to 6.9 × 10^8^ TCID_50_/mL) during 1 h of incubation in chaotropic salts (Figure 3B), which corresponded to half the increase compared to 48 hpi (Figure 3A,B). After 1 h of incubation, stabilization of the virus titers to about 3.5 × 10^8^ TCID_50_/mL was achieved. For all applied Tween 20 concentrations, except 0.05% and 0.5% Tween 20, the combination of chaotropic salts with Tween 20 led to a direct increase of infective MVA in the supernatant (Figure 3B,D).

Adding 0.01% Tween 20 led to the highest increase from all samples by 5.8 × 10^8^ TCID_50_/mL (from 3.9 × 10^8^ TCID_50_/mL to 9.7 × 10^8^ TCID_50_/mL), related to the control, but the initial rise resulted after 1 h of incubation to the same level of infectivity as the control (Figure 3B). All virus titers in the supernatant from 200× *g* centrifugation commuted after 1 h of incubation to infectivities between 2.7–3.7 × 10^8^ TCID_50_/mL. The highest loss of infective MVA particles during 1 h to 6 h of incubation in Tween 20 conditions was achieved for a detergent concentration of 0.5%, resulting in a reduction of 53%.

After 4600× *g* centrifugation, the highest MVA infectivities of the supernatant were achieved using 0.01% Tween 20 (Figure 3D). The virus titer increased by 27%, which is equal to 0.5 × 10^8^ TCID_50_/mL, compared to a control without Tween 20 addition up to 1 h after detergent addition. With prolonged incubation times up to 4 and 6 h, 0.01% and 0.05% Tween 20 showed virus titers equivalent to or higher than the control sample. The highest applied concentration of 0.5% Tween 20 led to an increase of 0.5 × 10^8^ TCID_50_/mL, even up to 1 h incubation time. The application of Tween 20 concentrations in a range of 0.1–0.5%, and exceeding 2 h of incubation, did not exceed the virus infectivity titers of the non-treated control (Figure 3D). The supernatant of the non-treated control from the 72 hpi harvest, resulting from a 6 h incubation time, resulted in a smaller range of virus infectivity with 0.4 × 10^8^ TCID_50_/mL, compared to TOH of 48 hpi, which had a span of 1.2 × 10^8^ TCID_50_/mL (Figure 3C,D).

The cell concentration at harvest 72 hpi was about 0.5 × 10^6^ cells/mL, higher (with a range of 3.7–4.4 × 10^6^ cells/mL) compared to 48 hpi (with a range of 3.3–3.9 × 10^6^ cells/mL, Figure 3E,F). The cell concentration of the control without Tween 20 decreased by roughly 1.9 × 10^6^ cells/mL (from 4.4 × 10^6^ cells/mL to 1.5 × 10^6^ cells/mL) during the incubation time in high ionic strength. This was a 0.9 × 10^6^ cells/mL higher difference compared to the final total cell concentration of the 48 hpi virus harvest. The Tween 20 concentration of 0.01% demonstrated a cell concentration of 2.5 × 10^6^ cells/mL, which was about 1.0 × 10^6^ cells/mL higher compared to the non-treated control (Figure 3F). Higher Tween 20 concentrations further decreased the cell concentration, with a minimum of 0.8 × 10^6^ cells/mL using 0.5% Tween 20. However, after 6 h of incubation, the cell disintegration with the addition of 0.5% Tween 20 resulted in 3.2 × 10^6^ cells/mL, whereas in the control without Tween 20, it resulted in 2.9 × 10^6^ cells/mL.

### 3.4. Clarification of Lysed Virus Harvest

**Figure 4:**
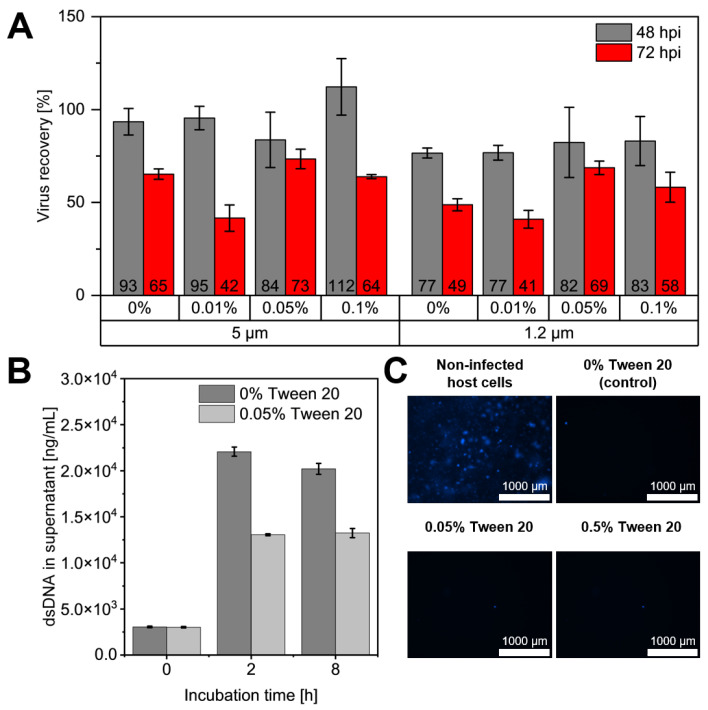
MVA-CR19.GFP clarification yields after detergent treatment and nuclei integrity. (**A**) Virus recoveries for clarification filtration of harvests from 48 hpi and 72 hpi, which were treated with different Tween 20 concentrations for 8 h. The harvests were clarified by a cascade of cellulose acetate syringe filters with 5 µm and 1.2 µm pore sizes. For the primary clarification, 6 mL of feed was used, and subsequently, 4 mL from the filtrate was used for the secondary filtration. The recoveries of 5 µm and 1.2 µm were related to the feed virus from primary clarification. Error bars represent standard deviations of technical triplicates. (**B**) Release of dsDNA to the cell culture supernatant of 48 hpi virus harvest, after 200× *g* centrifugation. Virus harvest was treated with 0% and 0.05% Tween 20 for 8 h. Error bars represent standard deviations of technical triplicates. (**C**) Hoechst 33,342 staining to determine the influence of high-salt environment and Tween 20 on the integrity of nuclei in a non-infected cell culture, 0% Tween 20, 0.05% Tween 20, and 0.5% Tween 20. Abbreviations: dsDNA, double-stranded DNA.

This experiment compares the MVA recoveries after the detergent treatment in the filtrate fractions from a filter cascade of 5 µm and 1.2 µm pore sizes. Since virus stability is important for a robust process, only Tween 20 concentrations up to 0.1% for the subsequent cell removal were considered. The clarification of 48 hpi virus harvest resulted in infective MVA recoveries of 84–112% using the 5 µm retention rate (Figure 4A). After secondary 1.2 µm filtration, the product recovery decreased to 77–83%. However, compared to the control without Tween 20, we achieved no increase in recoveries with the addition of Tween 20.

The clarification of 72 hpi virus harvest led to total MVA recoveries of 42–73% using a 5 µm retention rate, and 41–69% recovery after secondary 1.2 µm filtration. The lowest MVA recovery of 41% was achieved with 0.01% Tween 20 after both cascade steps. Simultaneously, the 0.01% Tween 20 fraction had a 77% higher residual cell concentration in the harvest compared to the control (Figure 3F). In summary, the recoveries from 48 hpi feed remained higher compared to 72 hpi material.

After detergent treatment, it was investigated if the remaining nuclei were intact to facilitate DNA removal during the clarification filtration. Therefore, the release of dsDNA into the 200× *g* supernatant of the control and the 0.05% Tween 20 sample at 48 hpi was measured (Figure 4B). The same initial amounts of dsDNA were detected in the supernatant for the control and the 0.05% Tween 20 sample, with 3041 ng/mL and 3009 ng/mL, respectively. After 2 h of incubation, the increasing factor of dsDNA in the supernatant was higher for the control, 7.3 versus 4.3. Prolonged incubation times until 8 h depicted a similar result.

In this experiment, we were also interested in live staining the DNA with the Hoechst 33,342 dye to evaluate the integrity of the nuclear membrane. In Figure 4C, a vital and non-infected cell culture showed blue spots, which represented stained nuclei as a positive control. The infected control, as well as the Tween 20 concentrations of 0.05% and 0.5%, showed no stained cell cores. Single blue spots were classified as an artefact.

### 3.5. Temperature and Detergent Concentration Effects on Virus Stability

**Figure 5:**
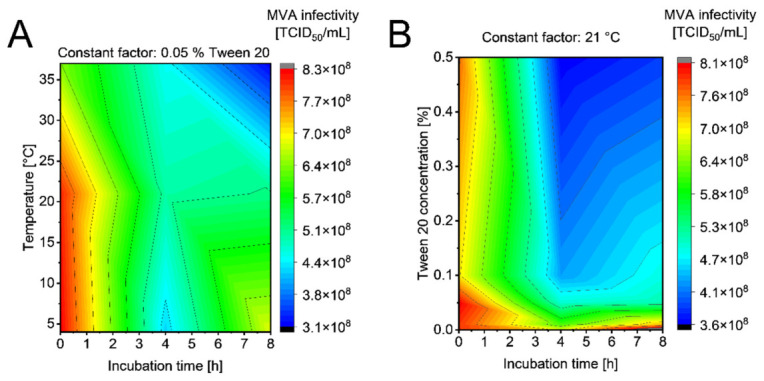
MVA-CR19.GFP infectivity stability in TCID_50_/mL from 48 hpi harvest, supplemented with 250 mM NaCl, 250 mM NaBr, and 150 mM KCl. The harvest was conditioned at different temperatures and concentrations of Tween 20. (**A**) Influence of temperature and incubation time at 0.05% Tween 20. (**B**) Effect of Tween 20 concentration and incubation time at 21 °C, measured in biological triplicates. Contour plots were created with OriginPro 2024b (OriginLab Corporation, Northampton, MA, USA).

The impact of process temperature on virus stability within a 48 hpi harvest was investigated to simulate conditions during downstream processing. At a constant concentration of 0.05% Tween 20, virus stability decreased as temperature increased (Figure 5A). Specifically, increasing the temperature from 4 °C to 37 °C resulted in a loss of 1.9 × 10^8^ TCID_50_/mL, with titers dropping from 8.3 × 10^8^ TCID_50_/mL to 6.4 × 10^8^ TCID_50_/mL. Over an 8 h incubation period, a decrease in virus titer was observed at all tested temperatures. The residual infectivity remaining in the harvest matrix after 8 h was 82% at 4 °C, 63% at 21 °C, and 48% at 37 °C.

The influence of Tween 20 concentration on infectivity was further examined at a constant temperature of 21 °C (Figure 5B). While 0.01% Tween 20 led to an overall loss of 8% of infectious MVA, higher concentrations resulted in a loss of infectivity that correlated with increasing detergent amounts. After 4 h of incubation, infectivity losses reached 38% with 0.05% Tween 20 and 53% with 0.5% Tween 20. Notably, following an initial reduction in infectivity, titers remained stable within the harvest matrix for the remaining observation period.

## 4. Discussion

Supply security for MVA-vectored therapies can be improved with intensified upstream bioreactor processes that increase total viral yields [50,51,52]. With regard to large-scale production, further optimization can be achieved with downstream processes that minimize overall product losses and maintain high specific activity.

The purification step with the highest MVA losses is the clarification step, as a significant fraction of vaccinia viruses remain cell-associated and are removed together with the cells or cellular debris [50,53]. This study examined the applicability of a detergent-based cell disruption for the release of intracellular MVA, and subsequently, its impact on clarification filtration to improve yields at the later process scale.

### 4.1. Detergent Selection and Viral Structural Integrity

First, we compared different detergents expected to be compatible with utilization in a vaccine manufacturing process, at concentrations below and above their CMC. The concentration must be close to or exceed the CMC in order to disrupt cell membranes [54]. The cell integrity was expected to decrease with later TOH, and milder treatment conditions can be realized with reduced amounts of detergent to achieve cell lysis.

The zwitterionic detergents Zwittergent 3–14 and CHAPS, as well as the ionic detergent SDC, were shown to be highly disruptive for the infected cells. A maximum cell reduction of 24% was obtained for Zwittergent 3–14. Even below the CMC, Zwittergent 3–14 and CHAPS showed a strong destabilizing effect on the infectious virus in the cell-free supernatant after low-speed centrifugation, resulting in reductions of 99% and 91%, respectively. Zwittergent 3–14 is reported in other studies to effectively solubilize outer membrane proteins which could also remove receptor and fusion apparatus membrane proteins from the virion surface [55,56].

We have tested SDC due to its anionic groups, with the rationale that the interaction with virus particles (that are also negatively charged) could be reduced [57]. However, SDC led to a 94% loss of virus infectivity before the CMC was reached. This observation is consistent with the literature, where a complete solubilization of the vaccinia core walls was described when using ionic detergents [56,58].

Although the non-ionic agents Tween 20 and Tween 80 appeared not to break up the cells within the tested incubation time of 1 h, the overall performance was favorable. While below the CMC of Tween 20, a loss of 16% of virus was noted; no loss of virus when Tween 80 crossed its CMC was observed. The maximal reduction of MVA infectivity was 91% for 0.5% Tween 20 and 36% for 0.05% Tween 80. In addition, for 0.005% Tween 80, an increase of 27% (roughly 2 × 10^8^ TCID_50_/mL) was observed.

The weak interaction of non-ionic detergents with virus particles facilitates their efficient removal via diafiltration or size-exclusion chromatography [33]. The effect of Tween 20 and Tween 80 is mild because they do not alter protein structures and have little impact on the lipid barrier. The literature for the application of polysorbates to MVA remains sparse, but for the related Orf-virus (ORFV, genus Parapoxvirus), it is reported that Tween 20 has no effect on the virus infectivity at a concentration of 0.05%. Tween 80 showed a slightly stabilizing effect with a concentration of 0.05% in a liquid formulation [25]. Similar to MVA, ORFV is investigated for therapeutic vaccination and has to be given at 0.5–1.0 × 10^8^ PFU per dose, demonstrating the importance of efficient DSP and formulation [3,59].

Consisting of a mainly lipophilic component with an 18-carbon oleate chain, Tween 80 remains embedded in lipid bilayers rather than transferring lipids into micelles [60,61]. For this reason, Tween 80 is reported as a stabilizing detergent for final formulations, with low disintegrating activity against lipid membranes, and most likely insufficient to promote the release of large virions such as MVA [18,25,61,62,63,64]. In comparison to the lipophilic Tween 80, the stronger hydrophilic character of Tween 20 allows a gentle cell lysis [37,65], and is reported to facilitate vaccine yields by increasing the membrane permeability [37,66]. We therefore selected Tween 20 as a lead candidate detergent in subsequent experiments.

### 4.2. Influence of Harvest Time

For the TOH 48 hpi and 72 hpi, it was already reported for a wildtype MVA that virus release to the supernatant increases, but total yields remain the same [67]. With 48 hpi the process time and aggregation effects of released virus particles were reduced. With a later TOH of 72 hpi, the process generated more stable virus titers, but cell disintegration and induction of apoptosis can promote the adsorption of host cell DNA to escaped virus particles [67].

As a challenging scenario for the detergent treatment, we tested different concentrations of Tween 20 at 48 hpi, when the cells are still engaged in the induced aggregates that facilitate cell-to-cell spread. In the default protocol, the release of infectious units into the supernatant is primarily mediated by the chaotropic salts (250 mM NaCl, 250 mM NaBr, 150 mM KCl) [40]. Addition of Tween 20 showed an increase of MVA in the supernatant up to 48%, and correlated with the detergent concentration. This facilitated release appears to be effective immediately after the addition of the detergent. Incubation times longer than 4 h using 0.01% and 0.05% Tween 20 were not superior to chaotropes, and rather lowered infectious titers, probably due to inactivation of viral particles. Detrimental effects appeared to be more profound for extracellular virus particles obtained by a high-speed centrifugation, compared to low-speed centrifugation, where fewer cellular components and debris that might protect against detergents are present.

The virus titers in the supernatant of the crude harvest at 72 hpi were four times higher than at 48 hpi, suggesting a release of virus particles by the progression of the cytopathic effect and disintegration of induced cell aggregates (Figure 3B) [67]. The difference in viral inactivation by using Tween 20 concentrations higher than 0.05% for the 72 hpi harvest is less than at 48 hpi. We assume that during the time from 48 hpi to 72 hpi the virus particles were released from host cells but formed flocs or aggregates with other particles that protect them from micellization [17]. Infectious activity in the supernatant was maintained from 0.01% to 0.1% Tween 20 within a 1 h incubation time. Higher detergent concentrations or incubation times exceeding 4 h had a negative impact, either on viral stability or by causing aggregation or even precipitation of micelles and enclosed virions. Due to the complex and polydisperse mixture of particles in the crude harvest, we did not attempt to determine particle size distribution (for example, by dynamic light scattering) to differentiate between inactivation and sequestration of infectious units.

### 4.3. The Impact of Detergent Treatment for Clarification Filtration

The impact of Tween 20 treatment on particularly challenging filtration processes was investigated to avoid losses during the clarification step. These losses arise from the relatively large size of MVA particles (250–350 nm) and from MVA fractions that remain cell-associated. After Tween 20 treatments, a cellulose acetate membrane with a 1.2 µm retention rate for filtration clarification demonstrated an improved MVA recovery, ranging from 77–83%, at a harvest of 48 hpi, as compared to the 72 hpi harvest, which yielded 41–69% (Section 3.4). Recoveries of 59.8–81.6% were reported from the supernatant of a batch and perfusion process with MVA-CR19.GFP when using polypropylene depth filters with a pore size of 0.45 µm [50]. Using 0.8 µm cellulose acetate filters, recoveries of up to 74% for clarification of vaccinia virus crude harvest (centrifuged cell lysate, and diluted 1:5 in 0.5 M ammonium sulfate and 3 M NaCl) were also reported [68]. The supernatant of a lysate from an adherent cell culture for the related Orf virus is reported with a recovery of 70–77% using either a polypropylene or cellulose-based material containing an inert filter aid in a two-stage clarification filtration [23]. Another study using combined 10 µm and 1.2 µm pore sizes of a polypropylene filter for poxvirus clarification resulted in about 75% Orf virus recovery for an unfrozen, crude harvest of a suspension cell culture [69]. Filtration after centrifugation using double-layer polypropylene filters with pore sizes of 0.8 + 0.45 µm for the smaller (non-enveloped) adenovirus was reported, with recoveries of 90% [70]. Further, polypropylene-based clarification of Influenza A virus with a 0.65 µm retention rate resulted in 85% product recovery [71]. The cellulose acetate material used here for clarification seems well suited for clarification of virus-containing supernatant and has a high chemical and mechanical stability with a low protein adsorption property [72,73]. The incubation at a salinity of 650 mM salt applied in this study may contribute to both the release of viral particles from cell debris into the supernatant during centrifugation-based clarification (Figure 3C,D) and high MVA recoveries of 77% and 49% following clarification by 1.2 µm filtration at 48 and 72 hpi harvests, respectively (Figure 4A).

Host cell-derived DNA is an important contaminant during viral vector processing that needs to be controlled to meet regulatory guidelines and to avoid detrimental viscosity of the lysate and DNA-tethered aggregation of virions during filtration steps. Determination of dsDNA is furthermore compounded by opposing effects. We suspect that the apparent rise of dsDNA concentration in the supernatant is an effect of improved accessibility to the Picogreen™ dye due to decondensation of DNA from the chromatin by high salinity [74]. However, surfactants such as non-ionic Tween 20 can also interact with complexes of cell-associated DNA and amphiphilic molecules to form micellar aggregates that sequester cell-associated DNA, thereby effectively masking detectable dsDNA in the supernatant (Figure 4B) [75,76]. For future process development, a salt-active endonuclease should be evaluated for host cell DNA removal at the increased salt level. For depletion of host cell DNA, the combination of 0.5% Tween 20 and 10 U/mL of the nuclease DENARASE^®^ (c-Lecta, Leipzig, Germany) for an AAV feed stream is reported, which indicates that detergents and nucleases are process compatible [27]. Gentle lysis that affects only the plasma membrane, without disrupting the nuclei, might also be an option. However, although detergents such as Nonidet-P40 can be used to extract intact nuclei containing the chromatin from cells [77], we were not able to permeabilize the plasma membrane in this study while keeping nuclei intact (Figure 4C).

### 4.4. Increasing Detergent Concentration and Temperatures Reduce MVA Infectivity

The CMC depends on both ionic strength and temperature [31,78,79]. To further assess critical process conditions, we kept the 0.05% Tween 20 environment constant but varied the temperature from 4–37 °C in the crude harvest (Figure 5A) [78,80,81]. This part of the study demonstrated that infectious activity is maintained (at 82% of the input activity) only at lower temperatures of 4 °C. At 37 °C, only 48% of the infectious units could be recovered. While the lowest tested detergent concentration of 0.01% Tween 20 led to the most stable MVA titers, this detergent concentration showed no beneficial clarification results compared to the control without surfactant (Figure 4A).

Among the limitations of our study are that we have not stringently fractionated our lysates to test the effects of cellular components, such as exosomes, membrane debris, released proteins, and nucleic acids, on the activity of the detergents. For example, although the virus particles are inactivated by Tween 20, a large fraction of 48% remained infective even under stringent 0.05% Tween 20, 37 °C, and 8 h of incubation. We speculate that the resistant fraction might be protected either within micellar structures, or by association with cellular debris, or even enclosed in non-functional intracellular compartments of infected cells (Figure 3E,F) [82]. Future studies might also benefit from a combination of detergents or an osmotic shock for cell lysis as an alternative strategy.

## 5. Conclusions

In summary, this study demonstrates that non-ionic detergents can be used to induce cell lysis while maintaining the infectious activity of enveloped MVA. Low concentrations of Tween 20 added at 48 hpi furthermore increased the release of infectious units out of the aggregates, induced for cell-to-cell transmission, into the cell-free supernatant. While an increase in supernatant virus titers was successfully demonstrated, contact times exceeding 4 h negatively impacted MVA infectivity. Therefore, in order to ensure robust process control, the use of a detergent is not recommended.

Due to the strong baseline effect of salts during detergent treatments, MVA recovery can be optimized by combining high ionic strengths in the crude harvest with a filtration step for subsequent clarification. The additional detergent treatment resulted only in a time-dependent increase in MVA yield and, in our hands, did not improve the overall yield.

## Figures and Tables

**Table 1:** Infective MVA particles in AGE1.CR.pIX culture supernatant (200× *g* centrifugation) at 48 hpi. Cells were treated with Tween 20 for less than 1 h, and the release was related to a control without Tween 20 treatment.

Tween 20 Concentration [%]	Release of Infective MVA [TCID_50_/mL]	Release of Infective MVA [%]
0.01	0.4 × 10^8^	9
0.05	0.5 × 10^8^	11
0.1	0.8 × 10^8^	17
0.25	0.8 × 10^8^	17
0.5	2.2 × 10^8^	48

## Data Availability

Data will be made available on request.

## Abbreviations

The following abbreviations are used in this manuscript:

DSP	Downstream Process
MVA	Modified Vaccinia virus Ankara
TOH	Time of harvest
